# Poly[(aceto­nitrile-κ*N*)-μ_3_-thio­cyanato-κ^3^ *N*:*S*:*S*-μ_2_-thio­cyanato-κ^2^ *N*:*S*-cadmium]

**DOI:** 10.1107/S1600536813015870

**Published:** 2013-06-15

**Authors:** Thorben Reinert, Inke Jess, Christian Näther

**Affiliations:** a Institut für Anorganische Chemie, Christian-Albrechts-Universität Kiel, Max-Eyth Strasse 2, D-24098 Kiel, Germany

## Abstract

The asymmetric unit of the title compound, [Cd(NCS)_2_(CH_3_CN)]_*n*_, consists of one Cd^II^ cation, two thio­cyanate anions and one aceto­nitrile ligand, all in general positions. The Cd^II^ cation is coordinated by three N atoms of two thio­cyanate anions and one aceto­nitrile ligand, as well as three S atoms of symmetry-related thio­cyanate anions within a slightly distorted octa­hedral coordination environment. The Cd^II^ cations are linked by μ-1,3(*N*,*S*) and μ-1,1,3(*S*,*S*,*N*) thio­cyanate anions into layers that are located in the *ab* plane.

## Related literature

For related structures, see: Wöhlert *et al.* (2011 ▶). For background to transition metal thio­cyanate coordination polymers and their magnetic properties, see: Boeckmann *et al.* (2010 ▶, 2011 ▶).
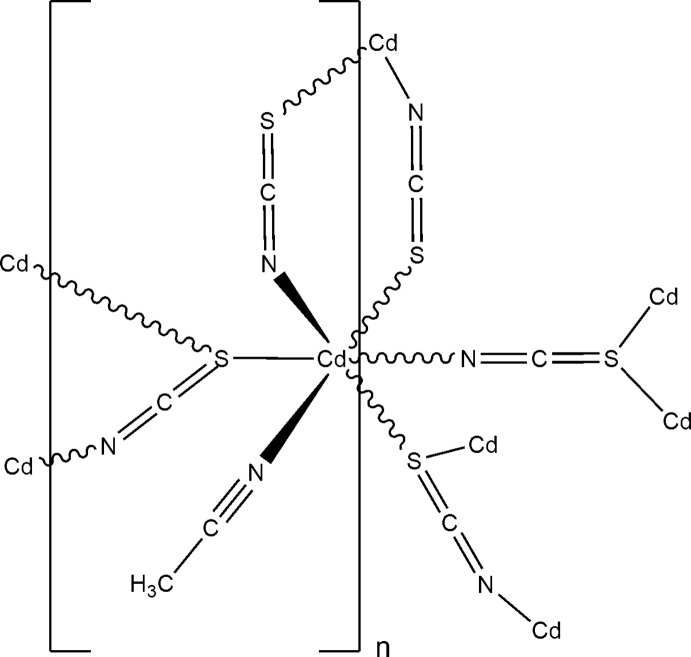


## Experimental

### 

#### Crystal data

[Cd(NCS)_2_(C_2_H_3_N)]
*M* *_r_* = 269.61
Orthorhombic, 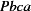

*a* = 13.0939 (7) Å
*b* = 8.9752 (5) Å
*c* = 14.2986 (11) Å
*V* = 1680.38 (18) Å^3^
*Z* = 8
Mo *K*α radiation
μ = 3.02 mm^−1^
*T* = 200 K
0.10 × 0.09 × 0.05 mm

#### Data collection

STOE IPDS-1 diffractometer
Absorption correction: numerical (*X-SHAPE* and *X-RED32*; Stoe & Cie, 2008 ▶) *T*_min_ = 0.447, *T*_max_ = 0.799
22741 measured reflections
2022 independent reflections
1943 reflections with *I* > 2σ(*I*)
*R*_int_ = 0.043

#### Refinement

*R*[*F* ^2^ > 2σ(*F* ^2^)] = 0.033
*wR*(*F* ^2^) = 0.090
*S* = 1.17
2022 reflections
93 parameters
H-atom parameters constrained
Δρ_max_ = 1.09 e Å^−3^
Δρ_min_ = −0.89 e Å^−3^

### 

Data collection: *X-AREA* (Stoe & Cie, 2008 ▶); cell refinement: *X-AREA*; data reduction: *X-AREA*; program(s) used to solve structure: *SHELXS92* (Sheldrick, 2008 ▶); program(s) used to refine structure: *SHELXL92* (Sheldrick, 2008 ▶); molecular graphics: *XP* in *SHELXTL* (Sheldrick, 2008 ▶) and *DIAMOND* (Brandenburg, 2011 ▶); software used to prepare material for publication: *SHELXTL* and *publCIF* (Westrip, 2010 ▶).

## Supplementary Material

Crystal structure: contains datablock(s) I, global. DOI: 10.1107/S1600536813015870/zl2553sup1.cif

Structure factors: contains datablock(s) I. DOI: 10.1107/S1600536813015870/zl2553Isup2.hkl

Additional supplementary materials:  crystallographic information; 3D view; checkCIF report

## Figures and Tables

**Table 1:** Selected bond lengths (Å)

Cd1—N2	2.254 (3)
Cd1—N1	2.287 (4)
Cd1—N11	2.340 (3)
Cd1—S2^i^	2.6253 (9)
Cd1—S1^ii^	2.7522 (8)
Cd1—S1^iii^	2.8780 (8)

Symmetry codes: (i) 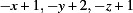; (ii) 
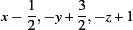
; (iii) 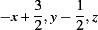.

**Fig. 1.**
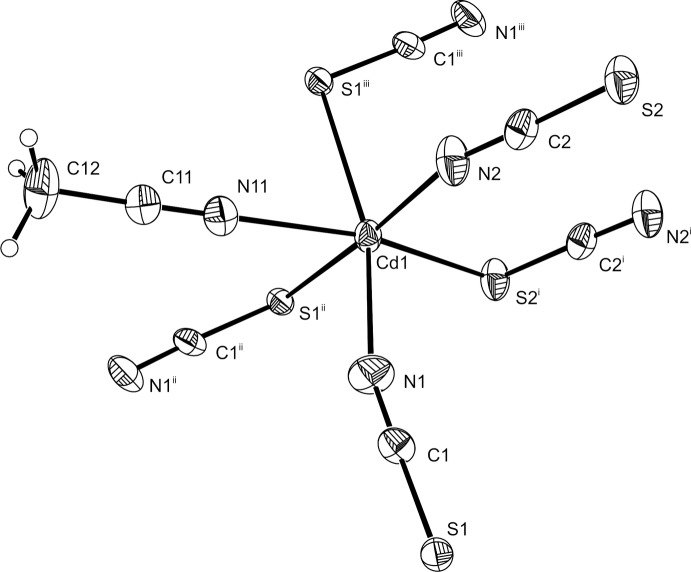
: Crystal structure of the title compound with labelling and displacement ellipsoids drawn at the 50% probability level. Symmetry codes: i = -x + 1, -y + 2, -z + 1; ii = x - 1/2, -y + 3/2, -z + 1; iii = -x + 3/2, y - 1/2, z; iv = x + 1/2, -y + 3/2, -z + 1; v = -x + 3/2, y + 1/2, z.

**Fig. 2.**
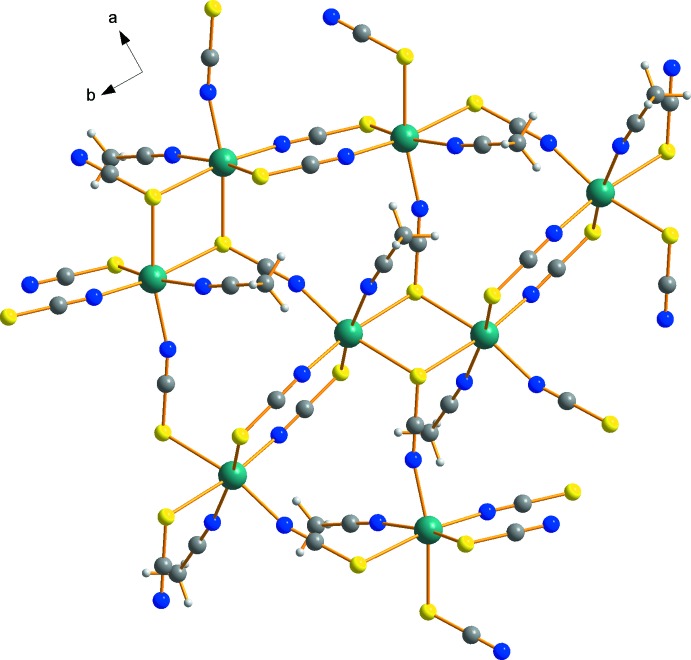
: Crystal structure of the title compound with view along the crystallographic c-axis.

## supplementary crystallographic information

### Comment

The structure determination of the title compound was performed as part of a project on the synthesis of new coordination polymers based on transition metal thiocyanates and the investigations on their magnetic properties (Boeckmann *et al.* (2010, 2011)). Within this project, we have reacted cadmium(II)thiocyanate with 4-*tert*-butylpyridine in acetonitrile, which resulted in the formation of crystals of the title compound by accident. In the crystal structure the Cd cations are surrounded by three N atoms of two *N*-bonded µ-1,3-briding thiocyanato anions and one acetonitril ligand as well as three S atoms of three *S*-bonded µ-1,1,3-bridging thiocyanato anions in a slightly distorted octahedral geometry (Fig. 1 and Tab. 1). The Cd···N distances range from 2.2544 (28) Å to 2.3396 (28) Å, the Cd···S distances from 2.6254 (9) Å to 2.8781 (8) Å (Table 1). The Cd cations are linked into dimeric units by pairs of µ-1,3-briding thiocyanato anions that are further connected into chains by single µ-1,3-briding anionic ligands. These chains are further connected by pairs of µ-1,1,3-bridging thiocyanato anions into layers which are parallel to the crystallographic *a*-*b*-plane.

### Experimental

The title compound was obtained accidently during the reaction of 68.6 mg Cd(NCS)_2_ (0.30 mmol) with 11.1 µ*L* 4-*tert*-butylpyridine (0.08 mmol) in 1.0 ml acetonitrile at RT in a closed 3 ml snap cap vial. After several months colourless blocks of the title compound were obtained.

### Refinement

The H atoms were positioned with idealized geometry, allowed to rotate but not to tip and were refined isotropic with *U*_iso_(H) = 1.5 *U*_eq_(C) of the parent atom using a riding model with C—H = 0.98 Å.

### Crystal data

**Table:**

[Cd(NCS)_2_(C_2_H_3_N)]	*F*(000) = 1024
*M**_r_* = 269.61	*D*_x_ = 2.131 Mg m^−^^3^
Orthorhombic, *P**b**c**a*	Mo *K*α radiation, λ = 0.71073 Å
Hall symbol: -P 2ac 2ab	Cell parameters from 22741 reflections
*a* = 13.0939 (7) Å	θ = 1.9–28.2°
*b* = 8.9752 (5) Å	µ = 3.02 mm^−^^1^
*c* = 14.2986 (11) Å	*T* = 200 K
*V* = 1680.38 (18) Å^3^	Block, colourless
*Z* = 8	0.10 × 0.09 × 0.05 mm

### Data collection

**Table:**

STOE IPDS-1 diffractometer	2022 independent reflections
Radiation source: fine-focus sealed tube	1943 reflections with *I* > 2σ(*I*)
Graphite monochromator	*R*_int_ = 0.043
Phi scans	θ_max_ = 28.1°, θ_min_ = 3.1°
Absorption correction: numerical (*X-SHAPE* and *X-RED32*; Stoe & Cie, 2008)	*h* = −17→17
*T*_min_ = 0.447, *T*_max_ = 0.799	*k* = −11→11
22741 measured reflections	*l* = −18→18

### Refinement

**Table:**

Refinement on *F*^2^	Secondary atom site location: difference Fourier map
Least-squares matrix: full	Hydrogen site location: inferred from neighbouring sites
*R*[*F*^2^ > 2σ(*F*^2^)] = 0.033	H-atom parameters constrained
*wR*(*F*^2^) = 0.090	*w* = 1/[σ^2^(*F*_o_^2^) + (0.0511*P*)^2^ + 2.768*P*] where *P* = (*F*_o_^2^ + 2*F*_c_^2^)/3
*S* = 1.17	(Δ/σ)_max_ = 0.001
2022 reflections	Δρ_max_ = 1.09 e Å^−^^3^
93 parameters	Δρ_min_ = −0.89 e Å^−^^3^
0 restraints	Extinction correction: *SHELXL92* (Sheldrick, 2008), Fc^*^=kFc[1+0.001xFc^2^λ^3^/sin(2θ)]^-1/4^
Primary atom site location: structure-invariant direct methods	Extinction coefficient: 0.0100 (7)

### Special details

**Table:**

Geometry. All e.s.d.'s (except the e.s.d. in the dihedral angle between two l.s. planes) are estimated using the full covariance matrix. The cell e.s.d.'s are taken into account individually in the estimation of e.s.d.'s in distances, angles and torsion angles; correlations between e.s.d.'s in cell parameters are only used when they are defined by crystal symmetry. An approximate (isotropic) treatment of cell e.s.d.'s is used for estimating e.s.d.'s involving l.s. planes.
Refinement. Refinement of *F*^2^ against ALL reflections. The weighted *R*-factor *wR* and goodness of fit *S* are based on *F*^2^, conventional *R*-factors *R* are based on *F*, with *F* set to zero for negative *F*^2^. The threshold expression of *F*^2^ > σ(*F*^2^) is used only for calculating *R*-factors(gt) *etc*. and is not relevant to the choice of reflections for refinement. *R*-factors based on *F*^2^ are statistically about twice as large as those based on *F*, and *R*- factors based on ALL data will be even larger.

### Fractional atomic coordinates and isotropic or equivalent isotropic displacement parameters (Å^2^)

**Table:**

	*x*	*y*	*z*	*U*_iso_*/*U*_eq_	
Cd1	0.580143 (17)	0.70475 (2)	0.516796 (17)	0.02222 (14)	
N1	0.7434 (3)	0.7780 (4)	0.4831 (2)	0.0369 (8)	
C1	0.8127 (2)	0.8423 (4)	0.4550 (2)	0.0248 (6)	
S1	0.91085 (5)	0.93385 (8)	0.40941 (5)	0.02174 (19)	
N2	0.5499 (3)	0.9136 (3)	0.5999 (2)	0.0360 (7)	
C2	0.5257 (2)	1.0352 (3)	0.6171 (2)	0.0264 (6)	
S2	0.49199 (8)	1.20580 (8)	0.64506 (6)	0.0318 (2)	
N11	0.6540 (2)	0.5645 (3)	0.6370 (2)	0.0298 (6)	
C11	0.6836 (2)	0.4805 (4)	0.6898 (2)	0.0271 (6)	
C12	0.7197 (4)	0.3726 (5)	0.7579 (3)	0.0470 (10)	
H12A	0.6824	0.2789	0.7500	0.070*	
H12B	0.7083	0.4113	0.8211	0.070*	
H12C	0.7929	0.3550	0.7484	0.070*	

### Atomic displacement parameters (Å^2^)

**Table:**

	*U*^11^	*U*^22^	*U*^33^	*U*^12^	*U*^13^	*U*^23^
Cd1	0.01867 (18)	0.01728 (18)	0.03070 (19)	−0.00145 (7)	−0.00221 (7)	0.00423 (7)
N1	0.0250 (16)	0.0377 (19)	0.048 (2)	−0.0124 (13)	0.0000 (13)	−0.0006 (13)
C1	0.0195 (13)	0.0244 (14)	0.0305 (14)	−0.0026 (12)	−0.0042 (11)	−0.0042 (12)
S1	0.0181 (3)	0.0197 (4)	0.0274 (4)	−0.0017 (2)	−0.0021 (2)	0.0012 (3)
N2	0.0481 (18)	0.0210 (13)	0.0388 (15)	0.0052 (12)	−0.0079 (14)	−0.0011 (12)
C2	0.0282 (14)	0.0249 (14)	0.0260 (13)	−0.0030 (12)	−0.0053 (11)	0.0051 (11)
S2	0.0458 (5)	0.0209 (4)	0.0285 (4)	0.0052 (3)	−0.0061 (3)	−0.0017 (3)
N11	0.0296 (13)	0.0277 (13)	0.0322 (13)	0.0014 (11)	−0.0037 (11)	0.0020 (11)
C11	0.0276 (14)	0.0254 (14)	0.0283 (14)	−0.0016 (12)	−0.0055 (12)	−0.0017 (12)
C12	0.053 (2)	0.039 (2)	0.049 (2)	−0.0052 (17)	−0.0246 (19)	0.0138 (17)

### Geometric parameters (Å, º)

**Table:**

Cd1—N2	2.254 (3)	S1—Cd1^v^	2.8780 (8)
Cd1—N1	2.287 (4)	N2—C2	1.163 (4)
Cd1—N11	2.340 (3)	C2—S2	1.643 (3)
Cd1—S2^i^	2.6253 (9)	S2—Cd1^i^	2.6253 (9)
Cd1—S1^ii^	2.7522 (8)	N11—C11	1.135 (4)
Cd1—S1^iii^	2.8780 (8)	C11—C12	1.452 (5)
N1—C1	1.148 (5)	C12—H12A	0.9800
C1—S1	1.659 (3)	C12—H12B	0.9800
S1—Cd1^iv^	2.7523 (8)	C12—H12C	0.9800
			
N2—Cd1—N1	92.08 (13)	N1—C1—S1	177.3 (3)
N2—Cd1—N11	97.64 (11)	C1—S1—Cd1^iv^	104.43 (11)
N1—Cd1—N11	85.58 (11)	C1—S1—Cd1^v^	103.92 (11)
N2—Cd1—S2^i^	98.45 (8)	Cd1^iv^—S1—Cd1^v^	98.29 (2)
N1—Cd1—S2^i^	93.60 (9)	C2—N2—Cd1	160.0 (3)
N11—Cd1—S2^i^	163.90 (7)	N2—C2—S2	178.1 (3)
N2—Cd1—S1^ii^	91.84 (9)	C2—S2—Cd1^i^	99.62 (11)
N1—Cd1—S1^ii^	164.41 (9)	C11—N11—Cd1	170.7 (3)
N11—Cd1—S1^ii^	78.95 (7)	N11—C11—C12	179.1 (4)
S2^i^—Cd1—S1^ii^	100.74 (3)	C11—C12—H12A	109.5
N2—Cd1—S1^iii^	172.22 (9)	C11—C12—H12B	109.5
N1—Cd1—S1^iii^	95.29 (9)	H12A—C12—H12B	109.5
N11—Cd1—S1^iii^	85.43 (7)	C11—C12—H12C	109.5
S2^i^—Cd1—S1^iii^	78.63 (2)	H12A—C12—H12C	109.5
S1^ii^—Cd1—S1^iii^	81.71 (2)	H12B—C12—H12C	109.5
C1—N1—Cd1	163.1 (3)		

Symmetry codes: (i) −*x*+1, −*y*+2, −*z*+1; (ii) *x*−1/2, −*y*+3/2, −*z*+1; (iii) −*x*+3/2, *y*−1/2, *z*; (iv) *x*+1/2, −*y*+3/2, −*z*+1; (v) −*x*+3/2, *y*+1/2, *z*.
